# Measurement of renal tumour and normal tissue perfusion using positron emission tomography in a phase II clinical trial of razoxane

**DOI:** 10.1038/sj.bjc.6601105

**Published:** 2003-07-15

**Authors:** H Anderson, J T Yap, P Wells, M P Miller, D Propper, P Price, A L Harris

**Affiliations:** 1 Cancer Research UK PET Oncology Group, Imperial College School of Medicine, Hammersmith Hospital, Du Cane Road, London W12 0NN, UK; 2 MRC Cyclotron Unit, Imperial College School of Medicine, Hammersmith Hospital, Du Cane Road, London W12 0NN, UK; 3 ICRF Medical Oncology Unit, Institute of Molecular Medicine, John Radcliffe Hospital, Oxford OX3 9DS, UK

**Keywords:** razoxane, positron emission tomography, antiangiogenic, tumour perfusion

## Abstract

Measurement of tumour and normal tissue perfusion *in vivo* in cancer patients will aid the clinical development of antiangiogenic and antivascular agents. We investigated the potential antiangiogenic effects of the drug razoxane by measuring the changes in parameters estimated from H_2_^15^O and C^15^O positron emission tomography (PET) to indicate alterations in vascular physiology. The study comprised 12 patients with primary or metastatic renal tumours >3 cm in diameter enrolled in a Phase II clinical trial of oral razoxane. Perfusion, fractional volume of distribution of water (VD) and blood volume (BV) were measured in tumour and normal tissue before and 4–8 weeks after treatment with 125 mg twice-daily razoxane. Renal tumour perfusion was variable but lower than normal tissue: mean 0.87 ml min^−1^ ml^−1^ (range 0.33–1.67) compared to renal parenchyma: mean 1.65 ml min^−1^ ml^−1^ (range 1.16–2.88). In eight patients, where parallel measurements were made during the same scan session, renal tumour perfusion was significantly lower than in normal kidney (*P*=0.0027). There was no statistically significant relationship between pretreatment perfusion and tumour size (*r*=0.32, *n*=13). In six patients scanned before and after razoxane administration, there was no statistically significant change in tumour perfusion: mean perfusion pretreatment was 0.81 ml min^−1^ ml^−1^ (range 0.46–1.26) and perfusion post-treatment was 0.72 ml min^−1^ ml^−1^ (range 0.51–1.15, *P*=0.15). Tumour VD and BV did not change significantly following treatment: mean pretreatment VD=0.66 (range 0.50–0.87), post-treatment VD=0.71 (range 0.63–0.82, *P*=0.22); pretreatment BV=0.18 ml ml^−1^ (range 0.10–0.25), post-treatment BV=0.167 ml ml^−1^ (range 0.091–0.24, *P*=0.55). Tumour perfusion, VD and BV did not change significantly with tumour progression. This study has shown that H_2_^15^O and C^15^O PET provide useful *in vivo* physiological measurements, that even highly angiogenic renal cancers have poor perfusion compared to surrounding normal tissue, and that PET can provide valuable information on the *in vivo* biology of angiogenesis in man and can assess the effects of antiangiogenic therapy.

Razoxane, also known as ICRF 159 ((±)-1,2-bis(3,5-dioxopiperazin-1-yl) propane), is a chemotherapeutic agent, which inhibits cell division in the premitotic and early mitotic phases of the cell cycle (Sharpe *et al*, 1970). Antitumour activity with razoxane has been reported in acute leukaemia, lymphomas, lymphosarcomas, colorectal cancer, and head and neck carcinoma (Hellmann and Burrage, 1969; Bakowski, 1976; Bellet *et al*, 1976; O'Connell *et al*, 1980). Inhibition of angiogenesis was noted in preclinical studies (Hellmann and Burrage, 1969; Salsbury *et al*, 1970, 1974; Le Serve and Hellmann, 1972). Hellmann and Burrage (1969) demonstrated inhibition of metastases in implanted Lewis lung carcinoma treated with razoxane. Salsbury *et al* (1970) found that the treated tumours were less hyperaemic on histological examination compared to controls, and instead of the network of poorly defined vascular channels, treated tumours contained only a few discrete blood vessels (Salsbury *et al*, 1970). Tumour vasculature studies using X-ray angiography and carbon black studies showed random, abnormal dilated vessels in untreated Lewis lung carcinomas. Treated tumour vessels appeared discrete with a regular vascular arrangement (Le Serve and Hellmann, 1972).

With the recent interest in chemotherapy agents with specific antiangiogenic effects, razoxane has been reinvestigated and has recently undergone a Phase II clinical trial in renal tumours (Braybrooke *et al*, 2000). Metastatic renal cell carcinomas are highly vascular, and neovascularisation due to tumour angiogenesis is a common feature (DeKernion, 1986; Sasaki, 1996). To provide a specific assessment of the potential antiangiogenic effect of razoxane in the Phase II trial, we used H_2_^15^O and C^15^O positron emission tomography (PET) to measure a range of vascular pharmacodynamic parameters.

Positron emission tomography is a noninvasive imaging modality capable of quantifying physiologic and metabolic processes *in vivo.* Techniques have been developed using ^15^O labelled water and carbon monoxide to quantify regional perfusion, fractional volume of distribution of water (VD) and blood volume (BV) within tissues. The background to PET measurements of vascular parameters is reviewed elsewhere (Anderson and Price, 2002). Briefly, the rationale behind their use in the clinical assessment of response to antiangiogenic agents is that the protracted administration (i.e. weeks rather than hours) of a drug that inhibits angiogenesis should lead to fewer blood vessels in tumours. Fewer new blood vessels should result in a reduction in perfusion (angiogenic areas of tumour are better perfused) and blood volume (fewer vessels less blood). Although VD is a nonvalidated parameter and has not yet been related to a physiological parameter, it is determined simultaneously with perfusion. Fractional volume of distribution of water is the proportion of the region of interest in which the radioactive water is distributed, for example, if VD=0.5, only half of the volume is perfused. Fractional volume of distribution of water is thought to represent the proportion of viable tissue within a region of interest (ROI; Anderson and Price, 2002). The PET techniques were originally developed to measure perfusion and related parameters in the brain and heart (Lammertsma *et al*, 1985; Araujo *et al*, 1991). They have subsequently been modified and used in tumours (Wilson *et al*, 1992). Although tumour blood flow and its modulation have been studied extensively in animal models, little work has been carried out using this technique to measure tumour perfusion in humans in order to evaluate therapeutic response. A small study using PET to measure perfusion in two patients with liver tumours before and after the administration of angiotensin II found a reduction in perfusion in normal tissue, but no change in the perfusion to tumours (Taniguchi *et al*, 1996). Recently, the results of PET measurements of tumour blood flow before and after administration of the antiangiogenic agent endostatin in a Phase I trial have been published (Herbst *et al*, 2002). In the endostatin trial, a statistically significant reduction in tumour blood flow was measured using H_2_^15^O PET 4 weeks after the start of treatment.

The aim of this study was to quantify perfusion, VD and BV in renal cell carcinoma *in vivo* before and after a course of razoxane in patients enrolled in a Phase II trial (Braybrooke *et al*, 2000). Antiangiogenic agents do not target established blood vessels and, therefore, are not expected to induce regressions in tumours. It is generally considered that protracted treatment will be required, and disease stabilisation is considered a relevant end point for assessing the clinical response of antiangiogenic agents. For these reasons, it was hypothesised that surrogate markers of response at the end of the first course of treatment (rather than hours after administration of a single dose) were most likely to measure drug-induced changes in vascular parameters. Based on the preclinical findings on the effect of razoxane in animal studies (Salsbury *et al*, 1970; Le Serve and Hellmann, 1972), our hypothesis was that following treatment with razoxane there would be a reduction in perfusion, VD and BV of tumours as the hyperaemic and abnormal vascular channels are replaced by fewer normal vessels.

## METHODS

### Patients

Patients were enrolled as part of a Phase II clinical trial of razoxane conducted at the ICRF Medical Oncology Unit, Oxford. Patient details are listed in Table 1

**Table 1:** Patient details

	**Renal tumour lesion imaged**	**Pretreatment tumour size^a^ (cm)**	**Prescan tumour**	**Postscan tumour**	**Spleen**	**Kidney**
Patient 1	Pelvic recurrence	12 × 14	X^b^	X		
Patient 2	Para-aortic nodes	4.6 × 2.7	X	X		
Patient 3	Primary renal mass	12.5 × 12	X			X
Patient 4	Para-aortic nodal recurrence	5 × 3.8	X	X		X
Patient 5	Primary renal mass	12.7 × 12.5	X	X	X	X
Patient 6	Lung metastasis	5 × 5	X	X		
Patient 7	Mesenteric mass	6 × 7	X	X	X	X
Patient 8	Primary renal mass	7.0 × 7.5	X		X	X
Patient 9a	Primary renal mass	7.5 × 9	X			X
9b	Para-aortic nodal recurrence	5 × 3.5	X			
Patient 10a	Primary renal mass	8 × 7.5	X			X
10b	Para-aortic nodal mass	5 × 4.5	X			
Patient 11	Primary renal mass	12 × 9	X	X		X
Patient 12	Mediastinal nodes	3 × 3	X			

a The pretreatment size of tumour was measured from the pretreatment CT scan by bidimensional measurements.

b X indicates that a particular tissue region could be analysed.

. Inclusion and exclusion criteria for the Phase II study are published elsewhere (Braybrooke *et al*, 2000). Patients referred for PET scans also had a tumour mass greater than 3 cm in diameter (to ensure adequate sampling of PET scan data) and had to agree to attend the Hammersmith Hospital on two separate occasions for PET scans. Permission to carry out the study was given by the Hammersmith Hospital NHS Trust Ethics Committee and the Central Oxfordshire Research Ethics Committee. The Administration of Radioactive Substances Advisory Committee gave authorisation for use of radionuclides. All patients gave written informed consent.

### Study design

Patients attended the Hammersmith Hospital as outpatients and were scanned prior to and after 4–8 weeks of treatment with daily oral razoxane (125 mg b.d.). Two measurements of perfusion and BV were recorded at each session with the patient in the same scanning position. Pretreatment measurements were compared with known tumour phenotype characteristics. Changes in perfusion, VD and BV after 4–8 weeks of treatment with razoxane were recorded and compared with clinical response criteria, which have been described in detail elsewhere (Braybrooke *et al*, 2000).

### Scanning protocol

Prior to scanning, patients were cannulated with an arterial line inserted into the radial artery. An i.v. line was inserted into an accessible vein in the other arm. An ECAT 931 08/12 PET scanner (CTI PET Systems, Knoxville, TN, USA) was used, which measures 15 transaxial planes in 0.65 cm slices covering a 10.8 cm axial field of view. Patients were positioned according to the location of their tumour on a CT scan, and the position recorded to use for the second scan to ensure that the pre- and post-therapy regions of interest for analysis were the same. A 20-min transmission scan using ^68^germanium was performed for attenuation correction. Patients then received a bolus infusion of H_2_^15^O at a dose of 600 MBq via the intravenous line over 20 s at a rate of 10 ml min^−1^ followed by a 2-min saline flush. A total of 28 frames of PET emission data were acquired (1 × 30 s, 1 × 20 s, 14 × 5 s, 3 × 10 s, 3 × 20 s, 6 × 30 s) starting approximately 30 s before the infusion. During this time, continuous arterial blood sampling was acquired using an online BGO detector and automated pump at a rate of 5 ml min^−1^ to measure the arterial input function (Ranicar *et al*, 1991). A discrete sample was also taken at the completion of the scan for cross-calibration of the continuous data. The patient positioning was maintained, and the H_2_^15^O infusion and data acquisition were then repeated after 15 min. For the C^15^O scans, C^15^O was inhaled through a loose fitting mask at a dose of 3 MBq ml^−1^ and at a rate of 500 ml min^−1^ for 6 min. Static images were then acquired at equilibrium 2 min after the end of C^15^O administration. Discrete blood samples were acquired at 0, 2, 4 and 6 min to measure the concentration of blood radioactivity. The C^15^O acquisition was repeated 10 min after the conclusion of the first scan. Emission and transmission data were reconstructed using a Hanning filter with a cutoff frequency of 0.5 U of the reciprocal of the sampling interval of the projection data resulting in an image resolution of 8.4 × 8.3 × 6.6 mm^3^ full-width at half-maximum at the centre of the field of view. Images were normalised for differences in detector efficiency, corrected for photon attenuation, calibrated to absolute units of radioactivity (kBq ml^−1^), and then transferred to a SUN SPARC Workstation for processing and analysis.

### Data analysis

**Figure 1:**
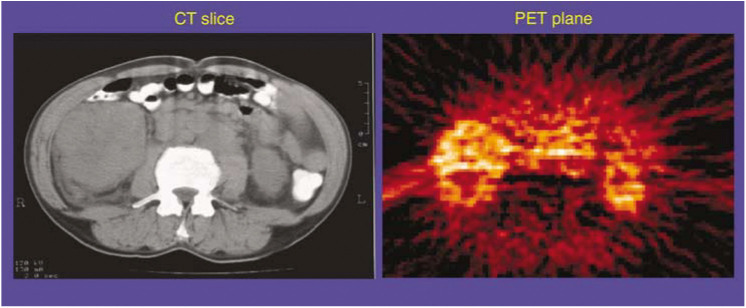
A single CT slice across the abdomen on the left and a single slice from a summed PET H_2_^15^O image on the right. Areas of increased brightness on the PET scan represent increased perfusion (the whiter the area, the higher the perfusion). There is a large right renal mass representing a primary renal cell carcinoma. This is seen on the PET image as the brighter area with a black centre representing a necrotic core. Vascular structures centrally, such as the aorta, also are bright.

Image visualisation and ROI analysis were performed using the Clinical Applications Programming Package (CAPP) software (CTI PET Systems, Knoxville, TN, USA). For each H_2_^15^O perfusion study, the dynamic images were summed over all time frames to generate signal-averaged ‘Add’ images, which enabled the anatomy to be visualised with improved signal-to-noise ratio. Since the C^15^O BV images are already summed static images, no further processing was required to improve visualisation. The blood flow Add images and BV images were compared with the pretreatment CT scan to aid the localisation of the tumour and other normal organs. Region of interests were then manually drawn on each transaxial plane of the PET blood flow Add images and/or BV images to identify the areas of tumour and normal tissue that were in the field of view. Many of the large renal cell masses had areas of necrosis seen as very low radiotracer accumulation. On the perfusion images, large central macroscopic areas of necrosis were avoided when drawing the ROIs, as these were considered to be nonviable tumour (Figure 1

). Since the patient positioning was maintained throughout each PET scanning sessions, the same ROIs could be applied to all the dynamic blood flow images and the BV images. The same ROIs were used for the pre- and post-therapy scan and checked by visual inspection to ensure that the same area was being analysed pre- and post-therapy.

For each ROI, the average measured radioactivity concentration of all pixels within the ROI was computed. In the case of the BV images, the mean concentration of each ROI was divided by the mean blood concentration obtained from discrete samples to determine the fractional BV within the ROI. In the case of the dynamic blood flow images, the averaged radioactivity in each ROI was calculated for each time frame to generate mean ROI time–activity curves. Nonlinear least-squares curve fitting of the ROI time–activity curves and the measured arterial input function was carried out using in-house software based on a modified version of the standard single-tissue compartmental model developed for the brain (Lammertsma *et al*, 1985). Parameter estimation of perfusion and VD were performed using fixed parameter values of delay and dispersion that were based on previous experiments using the given blood sampling rate and type and length of tubing. The delay and dispersion were adjusted manually to provide the best curve fits of the aorta or spleen and were then fixed for all other tissues. This resulted in the estimation of perfusion in ml min^−1^ ml^−1^ and VD for each tumour and normal tissue ROI sampled. The blood flow and BV scans were repeated in each session, and so the mean values were reported and used for all comparisons. The data for each type of ROI (e.g. tumour, kidney or spleen) were pooled and the pre- and post-treatment values were compared using a Student's paired *t*-test.

## RESULTS

In all, 12 of the Phase II trial patients were enrolled for the PET study (Table 1), and seven patients were scanned both before and after treatment with razoxane. Post-treatment scans were not available on five patients due to symptoms of progressive disease (four patients) and to reluctance to travel for a second scan (one patient). Of the 12 patients enrolled, eight metastases and six primary tumours were scanned (Table 1). The spleen was visibly definable in three of the patients and normal renal cortex was definable in eight patients. There were two technical failures of the repeat water scan (one patient) and C^15^O scan (one patient) in the same session. One patient (patient 2) had abnormally high values for tumour perfusion both pre- and post-treatment. The PET scan of this patient demonstrated a very high radiotracer concentration in the tumour mass. The tumour was adjacent to the right renal hilum. It is probable that the ROI around this tumour mass was contaminated by surrounding vascular structure, such as the renal artery or vein. As the model used to calculate perfusion in this study is not accurate at high flow values, the data for this patient were excluded from the analysis.

### Tumour vascular parameters

Pretreatment tumour vascular parameters varied between the 12 patients, with a mean perfusion of 0.87 ml min^−1^ ml^−1^ (range 0.33–1.67), mean VD of 0.74 (range 0.50–1.11) and mean BV of 0.18 (range 0.08–0.40). Of the six patients who had scans before and after treatment with razoxane (Table 2

**Table 2:** Vascular parameters in tumour and normal tissues

**Tissue**	Scanned*	**Mean perfusion (ml min^−1^ ml^−1^) (range)**	**Mean VD (range)**	**Mean BV (ml ml^−1^) (range)**
Tumour	Pre	0.81	0.66	0.18
		(0.46–1.26)	(0.50–0.87)	(0.10–0.25)
	Post	0.72	0.71	0.17
		(0.51–1.15)	(0.63–0.82)	(0.09–0.24)
Spleen	Pre	1.14	0.89	0.63
		(0.84–1.32)	(0.82–0.96)	(0.51–0.76)
	Post	1.28	0.77	0.54
		(0.94–1.62)	(0.77–0.78)	(0.54–0.55)
Kidney	Pre	1.84	0.69	0.17
		(1.18–2.88)	(0.52–0.78)	(0.11–0.20)
	Post	1.35	0.71	0.16
		(1.16–1.72)	(0.42–0.94)	(0.09–0.19)

**Figure 2:**
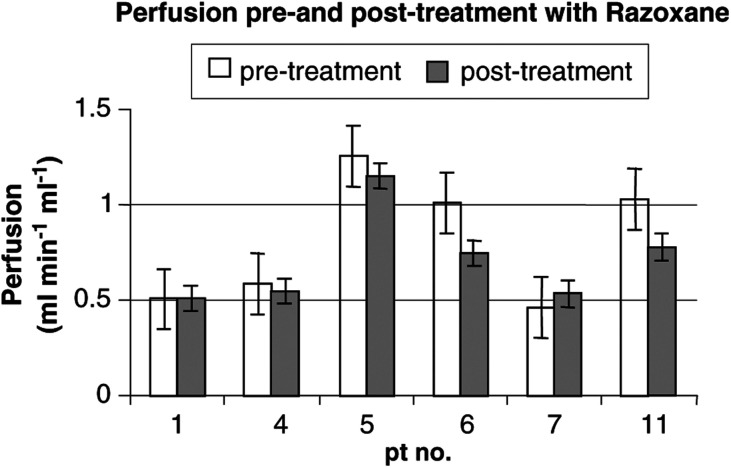
Graph showing perfusion in tumour masses before and after razoxane. Each value represents the mean of the two measurements made on each scanning occasion with standard error shown as error bars. There was no statistically significant difference in perfusion pre- and post-treatment with razoxane (*P*=0.15).

* Scans performed pre- and post-treatment with razoxane. VD=fractional volume of distribution; BV=blood volume. There was no statistical difference between pre- and post-treatment values for perfusion, VD and BV for tumour, spleen or kidney.

), the mean pretreatment perfusion was 0.81 ml min^−1^ ml^−1^ (range 0.46–1.26), while the mean post-treatment perfusion was 0.72 ml min^−1^ ml^−1^ (range 0.51–1.15). There was a reduction in perfusion following treatment in two out of six patients (Figure 2

), but this did not reach statistical significance (mean difference=0.09, *P*=0.15). The mean pretreatment BV was 0.18 ml ml^−1^ (range 0.10–0.25), while the mean post-treatment BV had virtually no change at 0.17 ml ml^−1^ (range 0.09–0.24, *P*=0.55). The mean pretreatment VD was 0.66 (range 0.50–0.87), while the mean post-treatment VD was 0.71 (range 0.63–0.82) with a slight increase that was not statistically significant (*P*=0.22).

### Relationship of perfusion to tumour size and response

**Figure 3:**
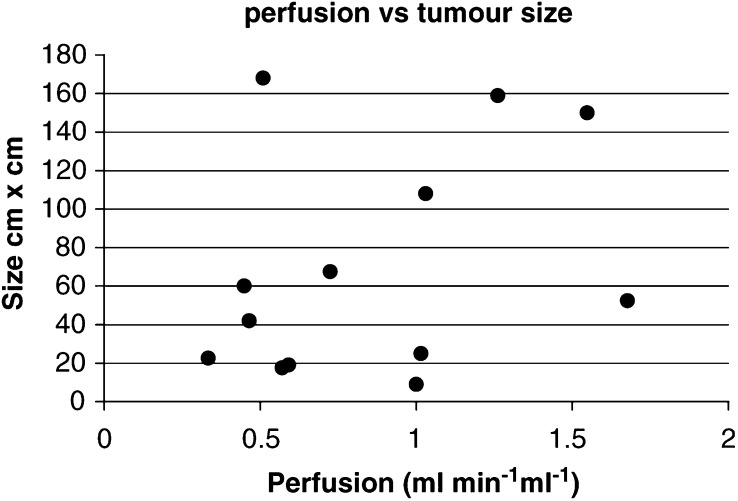
Lack of relationship between tumour size (bidimensional area measured on a CT scan) and tumour perfusion measured by PET (*r*=0.32, *n*=13).

In order to examine for any potential confounding influence of tumour size on vascular parameters, the relationship between perfusion and tumour size was assessed using the Spearman correlation coefficient. There was no statistically significant correlation between the pretreatment perfusion and size of a tumour as measured by a CT scan (*r*=0.32, *n*=13) (Figure 3

). The relationship of tumour perfusion with patient response and survival was also examined. Of the six patients who had scans before and after treatment, two patients had stable disease, and four had progressive disease. The analysis showed no association between pre- or post-treatment values, or change in perfusion following treatment, and clinical response at 8 weeks, or best overall response. There was no statistically significant correlation between pretreatment perfusion and patient survival following razoxane administration (*r*=0.18, *n*=11).

### Normal tissue perfusion

Table 1 documents which patients had normal tissue measurements performed, and the data are summarised in Table 2. Perfusion was lower in tumour compared to the other normal tissues studied. The perfusion of tumour and normal kidney was measured during the same scan session in eight patients (Table 3

**Table 3:** Tumour and normal kidney perfusion in patients who had both tissues measured in the same scan session

**Patient**	**Tumour perfusion (ml min^−1^ ml^−1^)**	**Renal perfusion (ml min^−1^ ml^−1^)**
3	1.55	2.10
4	0.59	1.35
5	1.26	2.85
7	0.46	1.50
8	1.68	1.55
9	0.72	1.70
10	0.45	1.75
1	1.03	1.65
		
Mean±s.d.	0.97±0.49	1.81±0.48*

* *P*=0.0027 for a paired *t*-test comparison of tumour and normal tissue data.

). A paired *t*-test comparison of the data showed that the perfusion of renal cancers was statistically significantly lower than that of normal kidney tissue (*P*=0.0027). In the four patients who had kidney perfusion measured both before and after treatment with razoxane, mean pretreatment values were 1.84 ml min^−1^ ml^−1^ (range: 1.18–2.88), while the mean post-treatment values were 1.35 ml min^−1^ ml^−1^ (range: 1.16–1.72). As for tumours, there was a reduction in post-treatment renal perfusion, but again this did not reach statistical significance (*P*=0.14). Pretreatment BV for renal cortex was similar to tumours with a mean value of 0.17 ml ml^−1^ (range 0.11–0.20) and a post-treatment mean BV of 0.16 ml ml^−1^ (range 0.09–0.19) with no significant change (*P*=0.14). Mean pretreatment VD was 0.69 (range 0.52–0.78), while there was no significant change in the post-treatment VD (mean=0.71, range 0.42–0.94, *P*=0.36).

Only two patients had both pre- and post-treatment measurements made in the spleen. Hence, there were insufficient numbers to perform a statistical analysis. For these two subjects, the pretreatment perfusion was 1.14 ml min^−1^ ml^−1^ (range 0.84–1.32), while the post-treatment perfusion was 1.28 ml min^−1^ ml^−1^ (range 0.94–1.62). Pretreatment BV was 0.63 ml ml^−1^ (range 0.51–0.76), while the post-treatment BV was 0.54 ml ml^−1^ (range 0.54–0.55).

## DISCUSSION

This is the one of the first reports of the measurement using PET of physiological vascular parameters (tumour perfusion, BV and VD) in patients to assess the effect of a potential antivascular**/**antiangiogenic agent in a Phase II study. Results have recently been reported of a Phase I study of endostatin that incorporated PET measurements of tumour blood flow (Herbst *et al*, 2002). In addition, we have measured vascular changes using PET in a Phase I study of the vascular targeting agent combretastatin A4 phosphate (Anderson *et al*, 2003). It should be noted, however, that the mechanism of action is different for antiangiogenic and antivascular agents. The former target the formation of new blood vessels and are expected to require protracted drug administration. The latter target the existing blood vessels of tumours causing extensive vascular shutdown and changes in vascular parameters within hours of administration (Anderson *et al*, 2003).

One difficulty of assessing the effect of antiangiogenic agents is that standard measures of tumour response based on volume change may be inappropriate, as tumour volume is not expected to change with response. Our hypothesis, based on preclinical data, was that a course of razoxane would reduce perfusion, BV and VD. However, we found that in the patients studied, there was no statistically significant change in vascular parameters after 4–8 weeks of razoxane therapy. Although the lack of control data and small sample size limit the conclusions that can be drawn from this study, one interpretation is that a single course of razoxane has no effect on tumour or normal tissue vasculature in the inoperable renal cell carcinomas studied.

Nevertheless, the observation of a reduction in perfusion in two out of six patients (Figure 2) is consistent with the expected razoxane-induced antiangiogenic effects. It is of interest to note that the decrease in perfusion was seen in two out of three patients with high pretreatment tumour perfusion and in zero out of three patients with low pretreatment perfusion. Whether any threshold effect is seen for razoxane remains to be established, and requires a larger study.

A number of observations have emerged from the study that may be useful for future pharmacodynamic studies of PET vascular parameters in clinical trials. In this study, renal tumour perfusion was statistically significantly lower than normal tissue perfusion. In contrast, perfusion in a series of breast tumours was consistently higher than in normal breast tissue (Mankoff *et al*, 2002). The lower perfusion in tumour *vs* normal kidney also contrasts with the observation that levels of the potent proangiogenic protein, vascular endothelial growth factor (VEGF), are 3–37 times greater in renal cancer compared to normal parenchyma (Brown *et al*, 1993). This high level of VEGF in renal tumours is due to constitutive upregulation because of the stabilisation of the key transcription factor hypoxia-inducible factor 1*α* (HIF-1*α*) (Ohh and Kaelin, 1999). Hypoxia-inducible factor 1*α* stabilisation results from mutations in the von Hippel–Lindau (VHL) gene, which occur in the majority of clear cell renal cancers (Meyer *et al*, 2000). The discrepancy of high angiogenesis but poor perfusion might result from a greater heterogeneity in the vascularity of tumour *vs* normal kidney. Tumours will contain not only ‘hot spots’ of angiogenesis (Vermeulen *et al*, 1996), but also poorly perfused areas that are hypoxic or necrotic. Perhaps future studies of PET vascular parameters in clinical trials of antiangiogenic agents could consider defining ROIs only in the most angiogenic areas of tumours.

Another observation from the study reported here is the lack of relationship between tumour size and pretreatment perfusion. Similarly, no statistically significant correlation was reported between blood flow and size for a series of advanced breast cancers (Mankoff *et al*, 2002). Any changes in tumour perfusion with increasing tumour size might act as a confounding influence on the ability to measure drug-induced changes in vascular parameters within clinical trials. The lack of relationship reported here and elsewhere suggests that inclusion of heterogeneously sized tumours will not be a limitation in the design of clinical trial of antiangiogenic agents involving PET measurements of vascular parameters in renal cell carcinoma. The lack of relationship found between tumour size and perfusion also suggests that the level of perfusion in a tumour might be fixed and under genetic rather than epigenetic control. This genetic control could relate to the mutational profile of each cancer and the ability of a tumour to produce angiogenic factors. The suggestion that the level of perfusion in a tumour is controlled genetically is also consistent with observations from studies of antiangiogenic therapy. For example, no reduction of tumour angiogenesis (measure as microvessel density) was found with the endostatin-induced shrinkage of an experimental human lung cancer model (Boehle *et al*, 2001).

A final observation from the study reported here is that pre- and post-treatment scans were obtained for only seven out of 12 (58%) of the patients enrolled for the study. This is lower than the 21 out of 22 (95%) described for a single-centre Phase I trial (Herbst *et al*, 2002). As our study involved patient travel to the PET centre, this observation has implications for the design of future clinical trials with adequate patient numbers, when patient travel between centres is required. The small sample size in our study limited the ability to demonstrate statistically significant razoxane-induced effects on vascular physiology. Recommendations for the design of future studies would be to include a sufficient number of patients to study responders and nonresponders as separate groups. Furthermore, because the effects of antiangiogenic therapy are relatively subtle and slow-occurring, there is a general need for additional control data to understand and quantify the changes that occur in tumours over time in the absence of treatment.

In summary, the work has shown the feasibility of measuring PET vascular parameters using H_2_^15^O and C^15^O in human renal cell carcinoma. New drugs are starting to enter clinical trials that specifically target tumour vasculature, and different methods are required to assess their effects. This study demonstrates that PET techniques for measuring physiological vascular parameters are a potentially useful tool in this area providing quantitative data in tumours and normal tissue. The short half-life of ^15^O allows multiple measurements within the same scanning session, which enables multiple assessments and the study of fast-acting therapies. The study highlights aspects of vascular physiology of tumours, which are of potential importance in monitoring new therapies and understanding tumour angiogenesis.
